# Expanded detection of *BAP1* alterations in cancer and tumor type-specific expression score comparison

**DOI:** 10.1101/2023.11.21.568094

**Published:** 2023-11-21

**Authors:** Ian R. Sturgill, Jesse R. Raab, Katherine A. Hoadley

**Affiliations:** 1 Bioinformatics and Computational Biology Curriculum, Department of Genetics, University of North Carolina at Chapel Hill, Chapel Hill, NC; 2 Department of Genetics, Lineberger Comprehensive Cancer Center, University of North Carolina at Chapel Hill, Chapel Hill, NC

**Keywords:** BAP1, gene expression profiling, genomic alteration, multiomics, tumor suppressor gene

## Abstract

*BAP1* is a tumor suppressor gene that was originally studied in uveal melanoma (UVM), kidney renal cell clear cell carcinoma (KIRC), and malignant mesothelioma (MESO). Early analyses focused on single-nucleotide variants, but other alteration types such as larger indels and gene-level copy number (CN) loss can also lead to loss of *BAP1* expression. We performed integrated multi-omic analyses using data from The Cancer Genome Atlas (TCGA) for 33 cancer types and more than 10,000 individuals. We combined and manually reviewed existing variant calls and new calls derived from a *de novo* local realignment pipeline across multiple independent variant callers including indel callers, increasing detection of high-quality somatic variant calls by 30% from 91 to 130, including 7 indels ≥40bp. Including CN loss alterations, 1561 samples from 32 cancer types were *BAP1*-altered, with alterations being predominantly CN-driven. Differential expression and survival analyses revealed both shared and tissue-specific consequences associated with *BAP1* alteration. Our findings broadly emphasize the improvements that are gained by using new computational approaches in large cancer-genome studies such as TCGA.

## Introduction

*BRCA* associated protein-1 (*BAP1*) has been identified as a tumor suppressor gene with activity across a broad range of biological processes^1–3^ including DNA damage repair^1,4,5^, cell cycle and cell proliferation^1,6–8^, and apoptosis^8–10^. This work led to further investigations into the role of *BAP1* mutations in promoting the development of cancer across multiple tissue types as well as the relation of *BAP1* loss to differential survival outcomes.^11–14^

*BAP1* mutations are most prevalent in uveal melanoma, mesothelioma, and renal cell carcinoma.^1,15^ Mutations occur very infrequently in other cancer types, often leading to exclusion from consideration due to underpowered analyses. However, among the cancer types that have been studied, consequences of *BAP1* loss are pleiotropic. It is therefore reasonable to assume that there is tissue-specific context that has not yet been systematically explored, presenting an opportunity to expand our understanding of the role of *BAP1* in cancer – particularly if we can incorporate additional cancer types into a larger set of analyses of *BAP1* alteration.

Although *BAP1* mutations have been studied in a small subset of cancer types^1,11–15^, there has been limited incorporation of other types of alterations that can lead to BAP1 loss. In mesothelioma, we demonstrated an increase of 40% of alterations leading to decreased *BAP1* expression by accounting for copy number loss and previously-undetected long indels.^16^ This suggests that many prior studies which focused more on single-nucleotide variants have missed important subsets of *BAP1*-altered samples, thereby impacting interpretation of the role of *BAP1*. Here, we account for these additional types of alterations across a larger set of cancer types represented in The Cancer Genome Atlas (TCGA) in order to better characterize functional consequences of *BAP1* alteration in cancer.

## Materials and Methods

### Somatic mutation calling workflow with *de novo* local realignment

10,414 pairs of tumor and matched-normal aligned binary alignment map (BAM) slice files of the *BAP1* locus and 100kb flanking regions from 33 cancer types were downloaded from The Cancer Genome Atlas (TCGA) Genomic Data Commons (GDC)’s harmonized hg38 mapped data using the command line GDC Data Transfer Tool v.1.6.1. The specific genomic region was hg38 chr3:52300003–52511030. Individual BAM slices were then processed through a Nextflow DSL 2 pipeline for somatic mutation calling. Briefly, slices were indexed using Samtools (version 1.9).^17^ Reads were realigned to the hg38 human reference genome (GRCh38.d1.vd1) using the ABRA2 (version 2.24–0) *de novo* local realigner with --undup and --no-edge-ci parameter flags.^18^ Somatic mutations were called using the Strelka2 somatic workflow (version 2.9.10) and Cadabra indel (version 2.24–0) variant callers after marking duplicate reads using bammarkduplicates from biobambam (version 2.0.87).^18–20^ Raw variant call format (VCF) output files were converted to mutation annotation format (MAF) using vcf2maf (version 1.6.21) and VEP (cache version 103).^21,22^

### Filtering, manual review, and characterization of somatic variants

Mutation calls were initially filtered based on individual variant caller quality tags (i.e., FILTER = PASS). These mutation calls were merged with calls from the GDC’s internal mutation calling pipeline consisting of four variant callers: MuSE, MuTect2, VarScan2, and pindel. Variants were further filtered to those that had variant allele frequency (VAF) ≥0.2 and mutant allele read count (t_alt_count) ≥2 followed by manual review using the Integrative Genomics Viewer (IGV) to further assess read support strength and evidence of any other confounding factors such as alignment at low-complexity regions and variant bases captured only by ends of reads.^23^ Current mutation calls for *BAP1* were then compared to historical calls generated for TCGA in the MC3 dataset and characterized by their somatic MAF variant classification.^24^ Final mutation calls were confirmed to have low expression within their tumor type, indicating impactful mutations. Samples with no corresponding tumor RNA sequencing data available were excluded from downstream analyses. Chromosomal start locations of mutations were plotted, together with variant classification information, on a lollipop plot adapted from cBioPortal’s MutationMapper web tool.^25,26^ Protein-level domain annotations and visualization were approximated and adapted from Haugh et al.^27^

### Definition of copy number loss

Estimates of focal gene-level copy number for *BAP1* and other genes were downloaded from the GDC, which used the ASCAT2 workflow to provide per-gene integer value estimations of gene copy numbers for each sample.^28^ Samples with gene-level copy number of less than two corresponded to copy number loss. Segment widths of samples with copy number loss were computed from segment start and end loci from masked copy number segment files from the GDC, which provided estimates of genomic windows of copy number loss and used the DNAcopy workflow.^29^ Plots of segments in relation to the chromosome were generated using plotgardener (version 1.4.2).^30^

### Differential mRNA expression and gene set enrichment analyses (GSEA)

Per-sample hg38 Spliced Transcripts Alignment to a Reference (STAR)-aligned unstranded counts files were downloaded from the GDC.^31^ Where appropriate, we adjusted for additional sources of variation (tumor purity, histological subtype, and/or previously-defined molecular subtype) in our model design in a cancer type-specific manner (Table S4). Thyroid cancer (THCA, n=505) had no detected altered samples and was excluded from differential expression analyses. Samples without sufficient subtype annotations and samples without tumor RNA sequencing data available were also excluded from analyses, leaving a total of 8146 samples. To partially account for potential effects of co-alteration of other chromosome 3p genes in instances of arm-level copy number loss, we filtered out non-*BAP1* chromosome 3p genes. *BAP1*-altered and -unaltered samples were then tested for differential gene expression using the DESeq2 R package (version 1.34.0).^32^ Briefly, we performed Wald testing with independent hypothesis weighting provided by the IHW R package (version 1.22.0).^33^

Initial results were then subjected to adaptive shrinkage estimation using the apeglm R package (version 1.16.0).^34^ Genes were annotated using biomaRt (version 2.50.3) via the annotables R package (version 0.1.91).^35–37^ Gene set and pathway analyses were conducted using the fgsea R package (version 1.20.0) and data from the hallmark and C8 gene sets from the MSigDB database.^38,39^ Comparisons of enrichment of hallmark pathways across cancer types focused on a list of hallmark pathways which were statistically significant in at least 5 individual cancer types. Volcano plots were generated using the EnhancedVolcano R package (version 1.12.0) and heatmaps were generated using the ComplexHeatmap R package (version 2.10.0).^40,41^

### Gene set and *BAP1* alteration signature scores

Apoptosis, DNA repair, EMT, *Notch* signaling, and oxidative phosphorylation gene set scores were computed separately within each tumor type from the MSigDB hallmark pathways as z-scores of per-sample sums of median-centered expression of all genes in each gene set. Samples within a tumor type with comparatively high expression of genes in the gene set will have higher sums and therefore higher z-score values. Within-tumor type *BAP1* alteration signatures were computed in the following way: differentially expressed upregulated in *BAP1*-altered samples were summed and multiplied by +1, while downregulated genes were summed and multiplied by −1. These per-sample values were summed, followed by z-score transformation. We compared the uveal melanoma (UVM)-derived *BAP1* alteration signature scores to within-tumor type *BAP1* alteration signatures of other cancer types using Spearman correlation. For the bile duct gene signature, gene sets BILE_DUCT_CELLS_1 through 4 from the MSigDB C8 cell type signature database were combined and samples were scored as above for the hallmark pathways.^39^ A specific *BAP1* mutation signature score was computed based on a gene set of differentially expressed genes between altered-with-mutation (Mut and Mut+CN) and copy number only-altered samples for five cancer types (CHOL, KIRC, LIHC, MESO, and UVM) with ≥5 mutations. We used the mclust (version 6.0.0) R package with default parameters to perform model-based clustering of mutation scores and classify samples as mutation “positive” or “negative”.^42^ For the 5-cancer type analysis, the cut-off for positive was a score of 237 and for the KIRC-only analysis, the cut-off was 138.

### Survival analysis

Curated survival data from Liu et al were downloaded from the TCGA PanCanAtlas Publications webpage (see Data Availability).^43^ Late-stage tumors classified as any stage 4 were excluded from downstream analyses. Tumors classified as any stage 0–2 were reclassified as low stage and tumors classified as any stage 3 were reclassified as high stage. For each cancer tumor type, we determined if *BAP1* alteration signature scores (either a positive or negative score) impacted progression-free interval (PFI). We implemented a maximum PFI cut-off based on median within-tumor type follow-up times to account for large differences in patient follow-up. Kaplan-Meier plots and corresponding risk tables were generated using the survival (version 3.5–5) and ggsurvfit^44,45^ Univariate Cox proportional hazards model results were computed for each tumor type, as well as multivariate results adjusting for tumor stage and subtype separately. For KIRC, we additionally evaluated survival using *BAP1* mutation signature scores.

## Data availability

### Whole exome sequencing BAM slice files

Because whole exome sequencing BAM files may contain personally identifiable information, they are subject to controlled access through the NIH database of Genotypes and Phenotypes (dbGaP, https://www.ncbi.nlm.nih.gov/gap/). Supplemental Table S1 contains a list of case and file IDs downloaded from the NIH Genome Data Commons repository (https://portal.gdc.cancer.gov/repository).

### Prior MC3 variant calls, tumor purity, and curated survival data

The following data were downloaded from the TCGA PanCanAtlas Publications webpage (https://gdc.cancer.gov/about-data/publications/pancanatlas):

- Mutations – mc3.v0.2.8.PUBLIC.maf.gz
- ABSOLUTE purity/ploidy file - TCGA_mastercalls.abs_tables_JSedit.fixed.txt
- TCGA-Clinical Data Resource (CDR) Outcome – TCGA-CDR-SupplementalTableS1.xlsx

### Tumor subtyping

Tumor subtype annotations of TCGA samples were compiled from many published sources. PubMed Central identifications (PMCIDs) are available per-sample in Supplemental Table S3B and per-cancer type in Supplemental Table S4A.

### Data availability

Nextflow code, BED files, and a Singularity container of software used for variant calling with ABRA2/Cadabra/Strelka2 are available on Zenodo (DOI: 10.5281/zenodo.10180501, https://zenodo.org/records/10180501). R code for generating all figures using data from the supplemental tables is also available on both Zenodo and the GitHub project repository for this manuscript (https://github.com/isturgill/Sturgill_2023_BAP1_Paper).

## Results

### *BAP1* somatic mutations in TCGA are underestimated and are predominantly deleterious

To determine the extent of underestimation of *BAP1* alterations via somatic mutation in TCGA and add occurrences of larger indels to existing mutation calls, we aligned BAM slice files containing the *BAP1* locus and 100kb flanking regions to the hg38 genome using a *de novo* local realignment approach with ABRA2 and two variant callers: Cadabra and Strelka2. These variant calls were merged with updated TCGA GDC calls which were also aligned to the hg38 genome and generated from four independent variant callers: MuSE, MuTect2, VarScan2, and Pindel. Out of 10,414 samples and 33 cancer types, a total of 1329 non-synonymous, non-intronic variants were detected in 981 individuals across the two updated hg38-aligned approaches with simple caller quality filtering (FILTER flag == “PASS”), including 14 indels ≥40bp in length (Table S2A). We compared this to an earlier iteration of variant calling used to generate the TCGA MC3 dataset, which included alignment to the hg19 genome and seven callers: MuTect, Varscan2, Indelocator, Pindel, SomaticSniper, RADIA, and MuSE.^24^ This earlier pipeline resulted in the detection of 243 variants from 217 individuals which similarly passed simple call quality filtering, none of which were ≥40bp (Table S2B).

**Figure 1.**
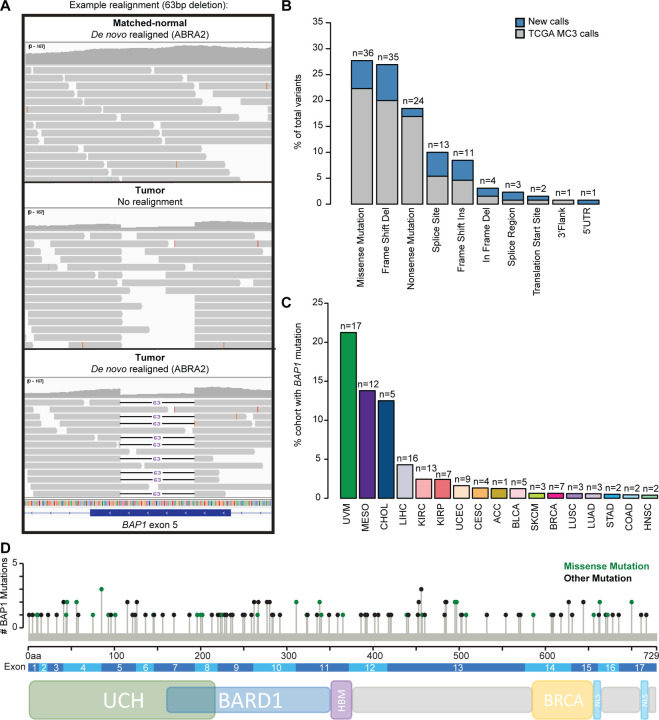
Prevalence and characterization of *BAP1* somatic mutations in TCGA pan-cancer tumor types. **(A)** Example *de novo* realignment with ABRA2 to the hg38 reference genome for a 63-bp indel detected in an LIHC tumor sample (TCGA-FV-A3I0) with visualization in IGV. **(B)** Distribution of *BAP1* somatic mutation types (total=130 variants). Blue, additional new mutations detected in the present workflow combined with updated TCGA GDC variant calls; grey, prior TCGA MC3 mutation calls. **(C)** Frequency of *BAP1* somatic mutations by TCGA tumor type. **(D)** Lollipop plot of *BAP1* somatic mutation locations across the length of the gene, adapted from cBioPortal^25,26^. Green, missense mutations; black, all other mutation types. Bottom: protein-level schematic of the major domains of interest, adapted from Haugh et al.^27^ UCH: ubiquitin carboxy-terminal hydrolase, BARD1: BARD1 binding domain, HBM: HCF binding motif, BRCA1: BRCA1 binding domain, NLS: nuclear localization signal.

To narrow down the new variant calls to those that impact *BAP1* expression, we required mutations to have low within-tumor type (cancer type-specific) expression, resulting in a reduction from 1329 to 445 variants in 280 individuals. Following further filtering and manual review of read support to identify high-quality mutation calls, we detected a final total of 130 variants for 111 *BAP1-*mutant samples across TCGA cancer types, including 7 larger indels ≥40bp that were not previously detected (Figure 1A, Supplemental Figure S1, and Supplemental Table S2C). When compared with similar filtering of the MC3 dataset, 39 (30%) were new calls and the remaining 91 were concordant (Figure 1B). All MC3 variants and mutant samples were captured in the new approach, with the exception of one variant, a 5bp deletion in KIRP sample TCGA-2Z-A9JD (Supplemental Tables S2C-S2D). However, our realignment approach detected two single-nucleotide variants (deletion and missense) in the similar region, suggesting a potential difference in hg19 versus hg38 alignment and downstream variant calling.

The majority of *BAP1* variants were deleterious nonsense and frameshift events (70/130, 53.8%), followed by missense mutations (36/130, 27.7%, Figure 1B and Supplemental Table S2C). Of the cancer types represented in TCGA, 17/33 (51.5%) have at least one *BAP1* mutant sample (Figure 1C). Only three tumor types had >5% mutation frequency: uveal melanoma (UVM), malignant mesothelioma (MESO), and cholangiocarcinoma (CHOL) (Supplemental Table S3A).

We did not identify strong hot spot loci and variants were broadly distributed across the length of the gene with no significant difference in protein-level domain representation for missense versus other mutation types (two-sided Fisher’s exact test p=0.42) (Figure 1D and Supplemental Table S2C). We observed 71/130 (54.6%) variants occurred at an annotated functional domain with 37/130 (28.5%) occurring at the catalytic UCH domain (Figure 1D and Supplemental Table S2C). Due to relatively small numbers of mutations, we were insufficiently powered to explore potential differences in impacts to specific functional domains.

### Copy number alterations constitute a major mechanism of *BAP1* loss across cancer types

**Figure 2.**
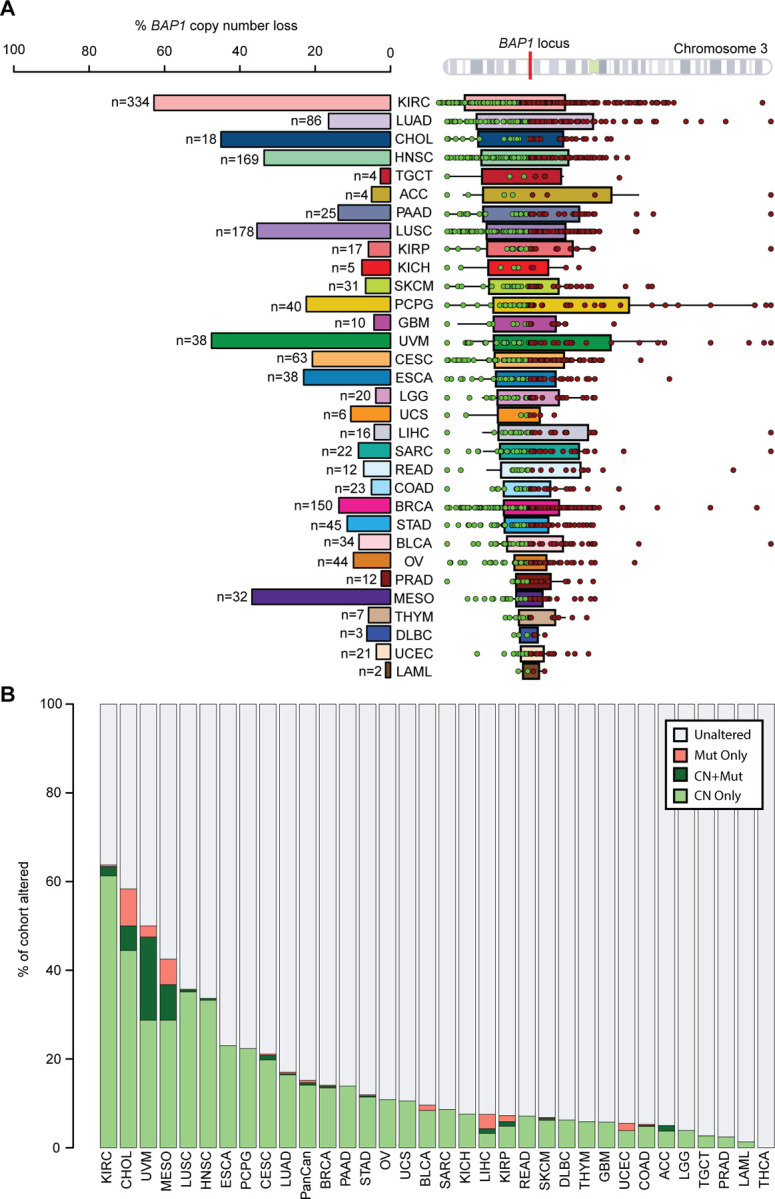
Copy number loss of *BAP1* is frequent in TCGA. **(A)** Left: percent and number of samples with *BAP1* gene-level copy number loss for each cancer tumor type. Right: segment widths of CN loss in samples with *BAP1* gene-level CN loss aligned against chromosome 3, which contains the indicated *BAP1* gene locus. One green circle and one red circle represent the start and end of a segment of loss for a single sample. Bars include the range of average segment starts and ends for each tumor type and lines extend out to represent upper and lower quartiles. **(B)** Percentage of each tumor type altered for *BAP1* by specific alteration types. Mut Only: somatic mutation, CN+Mut: co-occurring gene-level copy number loss and somatic mutation, CN Only: gene-level copy number loss only.

To assess the contribution of copy number (CN) alterations to a *BAP1* loss phenotype, we examined gene-level CN estimates of the *BAP1* gene. *BAP1* CN loss was observed in 1509 samples and estimated at single copy loss in 99.2% of all samples with CN loss (Table S3A-B). Gene-level CN loss occurs frequently (>30% of samples) in KIRC, UVM, CHOL, MESO, lung squamous cell carcinoma (LUSC), and head and neck squamous cancers (HNSC) (Figure 2A and Table S3A).

We also considered the CN segment widths around *BAP1* to determine if the loss is focal or arm-level. For many cancer types, the loss is arm-level, spanning large regions of the ~90Mb chromosome 3p arm on which *BAP1* is located and including regions corresponding to over 1000 annotated genes. For example, KIRC (median 58.3Mb, interquartile range IQR 32.9–80.4Mb), LUAD (median 61.0Mb, IQR 26.9–89.3Mb), and PCPG (median 51.7Mb, IQR 23.2–148.8Mb) have very large segment widths of loss (Table S3B). In contrast, MESO (median 9.9Mb, IQR 2.1–19.1Mb), PRAD (median 17.2Mb, IQR 4.5–33.7Mb), and UCEC (median 7.7Mb, IQR 4.8–16.6Mb) have relatively more focal *BAP1* CN loss, impacting an estimated 200–360 genes.

Combining CN and mutation data, 1561 samples from 32 tumor types were considered altered (Figure 2B and Supplemental Table S3A). Alterations are predominantly copy number-driven (1509/1561, 96.7%). Mutations were less frequent and co-occurring with estimated single-copy CN loss in 3.8% (59/1561) of samples and alone in 3.3% (52/1561). Regardless of tumor purity, mean variant allele frequency is 0.53 ± 0.22 (standard deviation) and does not differ between samples with mutations only and samples with both CN loss and mutations (two-sided Mann-Whitney U test with continuity correction p=0.29, Figure S2A). Variant allele frequency and tumor purity have a moderate positive correlation (linear regression Wherry adjusted r^2^=0.49, Figure S2B), suggesting that these mutations are less likely to be subclonal. The most highly-altered cancer types for *BAP1* are KIRC, CHOL, UVM, MESO, LUSC and HNSC – all with >30% altered samples within their tumor types (Figure 2B and Supplemental Table S3A-S3B). Among altered samples, there is also substantial variability in the mechanism of alteration. In KIRC and HNSC, alterations are primarily driven by instances of CN loss. However, in CHOL, UVM, MESO, and LIHC, mutations appear to play a much larger role. In the pan-cancer aggregate, all alteration types resulted in lower RNA-level expression of *BAP1* than unaltered samples (Figure S3 and Supplemental Table S3B).

### *BAP1* alteration is associated with distinct cancer type-specific expression signatures

To better understand the tissue and context-specific consequences of *BAP1* alteration, we examined differential gene expression across *BAP1*-altered and -unaltered samples by tumor type. We adjusted for additional sources of known variation such as tumor purity and subtype in our model designs in a cancer-specific manner (Table S4A). Of approximately 16,000 expressed protein coding genes tested in this manner, an average of 6% were significantly changed with a range from the lowest of 0.03% in LAML to the highest of 37.5% in UVM.

**Figure 3.**
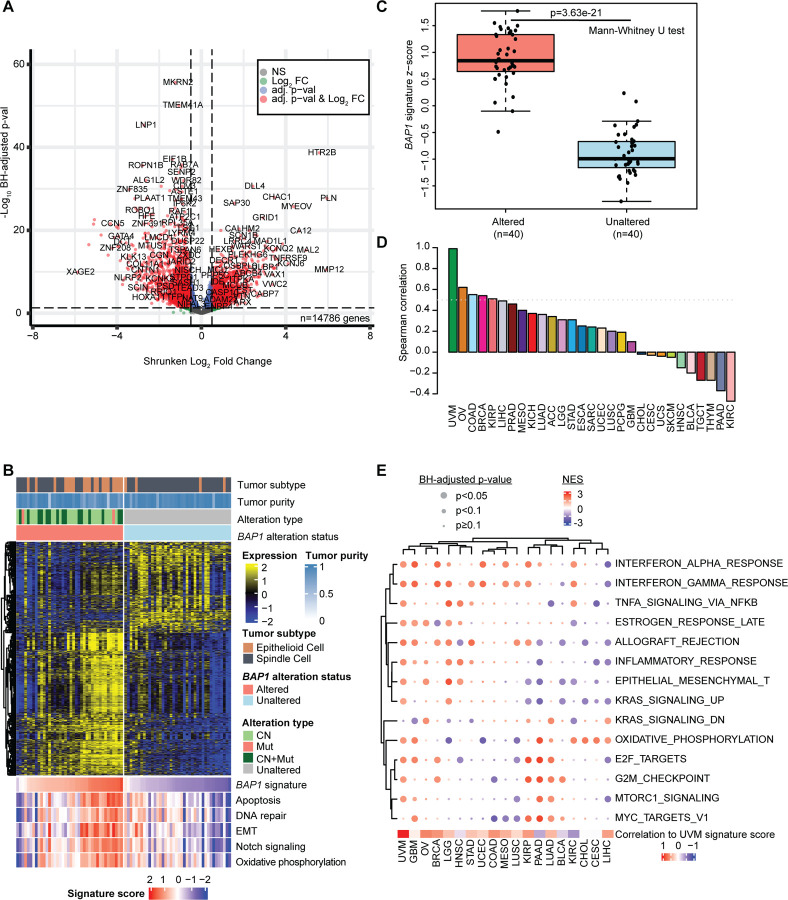
Transcriptional profiles of *BAP1* alteration. **(A)** Volcano plot of *BAP1* differentially expressed genes for uveal melanoma (UVM). Adjusted p-values are derived in DESeq2 using the Benjamini-Hochberg (BH) procedure. Log_2_ fold change values were shrunken using the apeglm R package. Red: adjusted p-value<0.05 and absolute log_2_ fold change≥1.5, blue: adjusted p-value<0.05, green: absolute log_2_ fold change≥1.5, grey: not significant (NS). **(B)** Heatmap of differentially expressed genes between *BAP1*-altered and -unaltered tumors and summarized geneset expression z-scores (bottom) in UVM with sample annotations. CN: gene-level copy number loss, Mut: mutation only, CN+Mut: gene-level copy number loss and mutation. Signature scores represent z-scores for the *BAP1* alteration signature as well as for MSigDB^39^ hallmark pathways at the bottom of the figure. **(C)** *BAP1* alteration signature scores in UVM separated by alteration status. P-value represents two-sided Mann-Whitney U test with continuity correction. **(D)** Spearman correlation values of the UVM *BAP1* altered signature with *BAP1* altered signatures derived in other TCGA tumor types. Correlations used complete observations. **(E)** Enrichment of hallmark pathways associated with *BAP1* alteration from gene set enrichment analyses and derived from differentially expressed genes between altered and unaltered samples. Circles show magnitude and significance of affected pathways, colored by normalized enrichment score (NES) and with three sizes of circles representing Benjamini-Hochberg adjusted p-values, large: p<0.05; medium: p<0.1; small: p≥0.1.

UVM exhibited particularly strong RNA expression patterns that distinguish *BAP1*-altered tumors with 5541 differentially expressed genes (Figure 3A). The CN region lost in UVM encompassed a large amount of chromosome 3p; therefore, we filtered out these genes for visualization and downstream analyses (Figure 3B). We computed per-sample signature z-scores for *BAP1* alteration based on this set of genes. A positive score represents a phenotype more similar overall to *BAP1*-altered samples while a negative score is more unaltered-like. Expression z-scores were similarly generated and visualized for major MSigDB^39^ hallmark genesets with prior implications for *BAP1*. In UVM, *BAP1* alteration signature scores were positively correlated with these geneset scores: apoptosis (Spearman ρ=0.65), DNA repair (Spearman ρ=0.51), EMT (Spearman ρ=0.65), *Notch* signaling (Spearman ρ=0.71) and oxidative phosphorylation (Spearman ρ=0.59) (Figure 3B and Supplemental Table S4B). 38/40 (95%) *BAP1*-altered UVM samples had a positive *BAP1* alteration signature score, indicating that altered samples are more similar to each other despite some observed heterogeneity (Figure 3C).

To investigate whether there is shared biological signal in response to *BAP1* alteration across cancer types, we compared UVM-derived signature scores with signatures derived from the other cancer types (Figure 3D). For most cancer types (66%, 19/29), BAP1 alteration scores were positively correlated, suggesting that the scores capture shared variation in gene expression due to *BAP1* alteration. However, 10 tumor types had negative correlations, including KIRC and further highlighting that although there may be shared consequences of *BAP1* alteration, those consequences are not universal. Consequences of *BAP1* alteration among all hallmark gene sets were cancer type-specific (Figure 3E). Pathway analysis of the most frequently differentially expressed gene sets across cancer types showed enrichment in immune response (18 tumor types), epithelial-mesenchymal transition (EMT, 8 tumor types), oxidative phosphorylation (10 tumor types), and proliferation (15 tumor types)– all known roles of *BAP1*. Clustering the normalized enrichment scores for the tumor types showed two main branches (Figure 3E). The left branch included tumor types with *BAP1* altered signatures with generally higher similarity to UVM (Figures 3D and bottom of 3E) and generally showed normalized enrichment score (NES) values in a similar direction. The right branch were tumor types with *BAP1* altered signature scores with less similarity to UVM and showed opposite enrichment of several pathways. One example is EMT where HNSC, LGG (low grade glioma), OV (ovarian carcinoma), and UVM tumor types exhibited upregulated expression of EMT pathway genes. In contrast, in the renal cancers KIRC and KIRP, pancreatic adenocarcinoma (PAAD) and urothelial bladder carcinoma (BLCA), *BAP1* alteration was associated with decreased expression of genes in these sets.

### *BAP1* alteration characterizes a molecularly distinct subset of hepatocellular carcinomas

**Figure 4.**
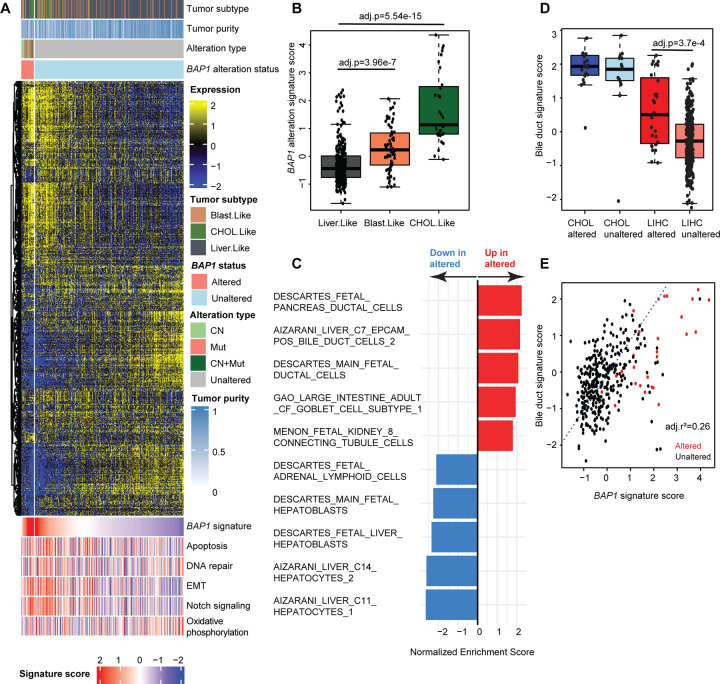
Alteration of *BAP1* in hepatocellular carcinoma (LIHC) is associated with a more duct-like phenotype. **(A)** Heatmap of differentially expressed genes between *BAP1*-altered and - unaltered tumors and summarized geneset expression z-scores (bottom) in LIHC with sample annotations. CN: gene-level copy number loss, Mut: mutation only, CN+Mut: gene-level copy number loss and mutation. Signature scores represent z-scores for the *BAP1* alteration signature as well as for MSigDB^39^ hallmark pathways at the bottom of the figure. **(B)** LIHC *BAP1* alteration signature scores separated by molecular subtype from Damrauer et al.^14^ P-values are derived from pairwise two-sided Mann-Whitney U tests with continuity correction and Bonferroni adjustment (adj.p). **(C)** Top five differential cell type signatures from the MSigDB C8 gene set collection in both directions between *BAP1*-altered and -unaltered LIHC tumors. Shown results are statistically significant with Benjamini-Hochberg adjustment. Red: up-regulated in *BAP1*-altered samples, blue: downregulated in *BAP1*-altered samples. **(D)** Specific comparison between CHOL and LIHC *BAP1*-altered and -unaltered samples of bile duct gene expression from four combined signatures from the MSigDB C8 gene set collection. Adjusted p-values are derived from pairwise two-sided Mann-Whitney U tests with continuity correction and Bonferroni adjustment (adj.p). **(E)** Linear regression showing correlation of *BAP1* and bile duct signature scores with Wherry adjusted r^2^ (adj.r^2^). Red points indicate *BAP1*-altered samples. Dotted line represents identity (y=x) line.

Because *BAP1* alterations have been previously shown to constitute a molecularly distinct subset of tumors in LIHC and may play a unique role in regulating cell identity and differentiation processes in the liver, we selected LIHC for more focused analyses.^14,46,47^ In LIHC, tumors carrying *BAP1* alterations were distinct from unaltered samples and within-tumor type alteration signature scores were consistently positive (Figure 4A). Similar to UVM, *BAP1*-altered samples in LIHC were positively correlated with apoptosis (Spearman ρ=0.23), EMT (Spearman ρ=0.44), and *Notch* signaling (Spearman ρ=0.43) (Supplemental Table S5A). Annotations of tumor molecular subtyping from Damrauer et al. revealed that this altered subset was enriched for blast-like and cholangiocarcinoma-like LIHC samples.^14^ These two molecular subtypes were also observed among the unaltered samples (77/330, 23.3%). Blast-like and cholangiocarcinoma-like tumors also have *BAP1* alteration signature scores higher than the other LIHC tumors (Figures 4A-B and Supplemental Table S3B, pairwise two-sided Mann-Whitney U test with continuity correction and Bonferroni adjusted p=3.96e-7 and p=5.54e-15 respectively). To further explore this relationship, we performed gene set enrichment analysis using gene sets published from single cell experiments represented in the MSigDB C8 database (Figure 4C and Supplemental Table S5B).^39^ The top five upregulated cell signatures in *BAP1*-altered samples consisted predominantly of ductal phenotypes, a characteristic of cholangiocarcinoma.^48^ In contrast, the top five downregulated cell signatures included mature hepatocytes and hepatoblasts.

We examined expression of bile duct cell markers by computing bile duct signature scores based on four combined bile duct cell signature gene sets from the MSigDB C8 database. We used CHOL samples as a comparison and observed that LIHC samples with *BAP1* alterations were enriched for bile duct markers relative to unaltered samples (Figure 4D and Supplemental Table S5A, pairwise two-sided Mann-Whitney U test with continuity correction and Bonferroni adjusted p=3.7e-4). Further, bile duct signature scores in LIHC tumors had a small positive correlation to *BAP1* alteration signature scores (Figure 4E and Supplemental Table S5A, linear regression Wherry adjusted r^2^=0.26), suggesting that the signature captures expression of some genes that separate cholangiocyte and hepatocyte identity.

### Signatures for *BAP1* alteration are associated with differential survival outcomes

**Figure 5.**
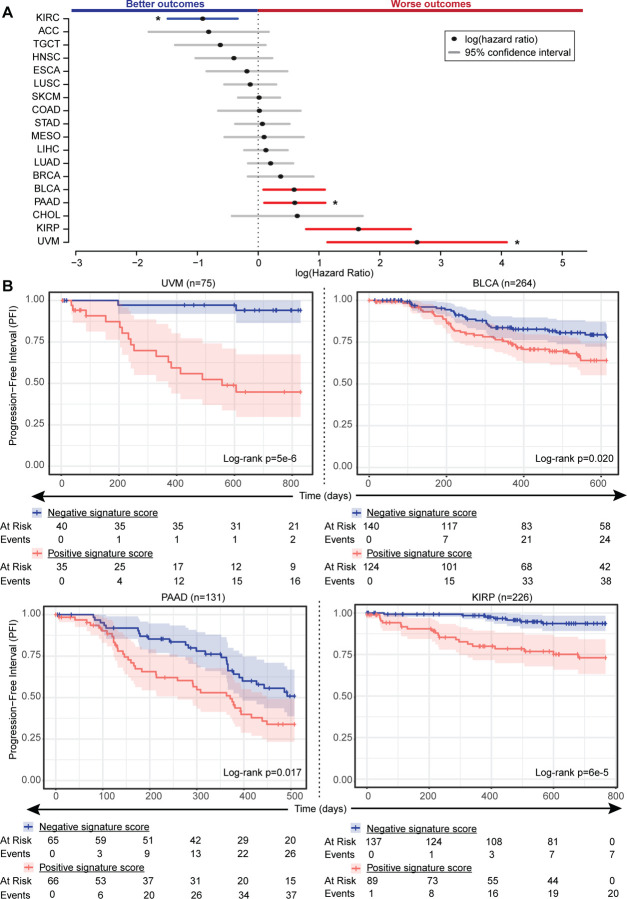
*BAP1* alteration is associated with worse progression-free survival outcomes in multiple cancer types. **(A)** Plot of univariate Cox proportional hazards ratios (HRs) as points together with their corresponding 95% confidence intervals (C.I.) as lines, comparing positive versus negative within-tumor type *BAP1* alteration signature scores. Red: worse outcomes with positive HRs, blue: better outcomes with negative HRs. Asteriks denote cancer types which retained significance in multivariate models adjusting for both tumor stage and subtype. **(B)** Kaplan-Meier curves showing progression-free survival for three tumor types with positive HR. Included statistics are logrank p-values. Below each set of curves is a corresponding risk table for each time point.

To evaluate whether changes in gene expression associated with *BAP1* alteration also had clinical impact, we assessed progression-free survival for each tumor type on the basis of *BAP1* alteration signature scores using Cox proportional hazards models. In univariate models, positive scores were associated with worse progression-free survival outcomes in four tumor types: UVM (hazard ratio HR=13.64, p=5.1e-4), KIRP (HR=5.2, p=1.8e-4), BLCA (HR=1.8, p=0.022), PAAD (HR=1.83, p=0.019), and improved outcomes in KIRC (HR=0.4, p=1.9e-3) (Figure 5A and Table S6A). Only three tumor types retained significance when adjusting separately for tumor stage and subtype: UVM, PAAD, and KIRC. BLCA was at the threshold of significance when accounting for molecular subtype (p=0.05, Supplemental Tables S5B-S5C). Progression-free Kaplan-Meier survival curves were generated for the univariate models and censored at within-tumor type median follow-up times for the four tumor types with worse survival outcomes: UVM, BLCA, and PAAD, and KIRP (Figure 5B). The clear separation of survival curves for this small subset of tumor types suggests that the *BAP1* alteration signature score captures tissue-specific gene expression changes that are survival-relevant.

### Characterization of *BAP1* mutation-driven alterations

**Figure 6.**
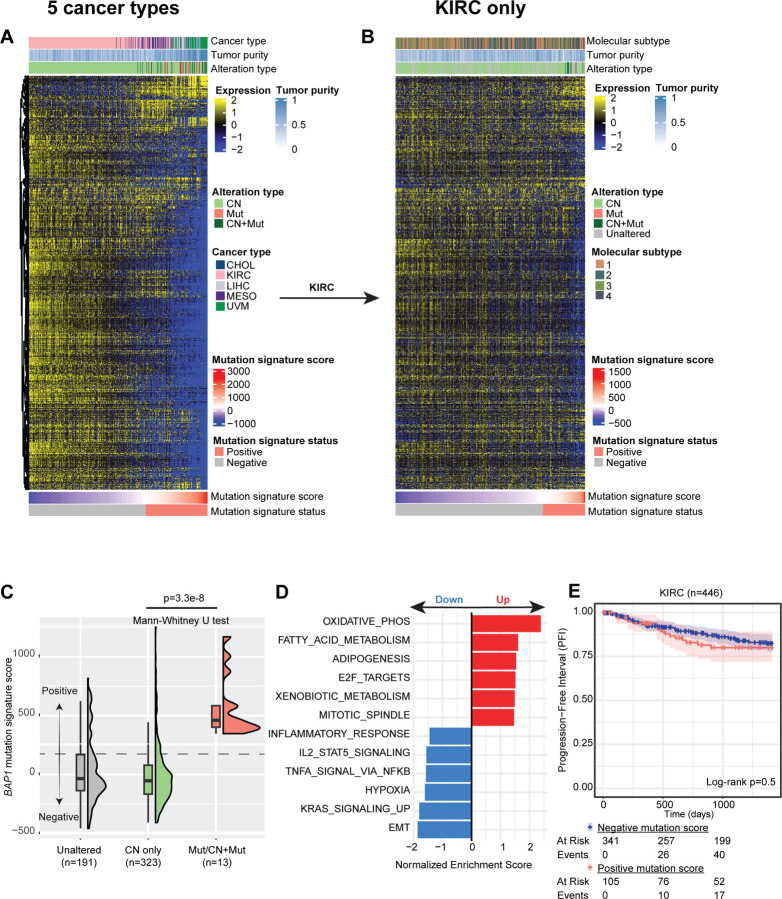
Characterization of *BAP1* mutation-driven alterations. **(A)** Heatmap of differentially expressed genes within *BAP1*-altered between samples with mutations (Mut and CN+Mut) versus samples with CN loss alone (CN) in cancer types with higher representation of mutations (CHOL, LIHC, MESO, UVM, and KIRC). CN: gene-level copy number loss, Mut: mutation only, CN+Mut: gene-level copy number loss and mutation. Mutation signature scores and status were derived from these differentially expressed genes using the approach detailed in the Methods section. **(B)** Heatmap of expression of genes from A for all KIRC samples, including both *BAP1-*altered and -unaltered samples. Molecular subtypes represent mRNA-based clusters from PMC3771322. **(C)** Distribution of *BAP1* mutation signature scores across KIRC alteration types. P-value is derived from two-sided Mann-Whitney U test with continuity correction. **(D)** Normalized enrichment scores for MSigDB hallmark gene sets derived from genes differentially expressed in CN-driven alterations versus mutation-driven alterations in KIRC. Red: up-regulated in mutation-driven alterations, blue: down-regulated in mutation-driven alterations. **(E)** Kaplan-Meier curve showing univariate progression-free survival for KIRC stratified by positive versus negative *BAP1* mutation signature scores. Included statistic is logrank p-value and the corresponding risk table for each time point is also below.

The KIRC samples showed different patterns for both gene expression and survival compared to other cancer types. Of altered tumors, KIRC was almost entirely driven by copy number loss (99%) and alteration status was associated with improved outcomes. We sought to better understand the specific biological consequences the type of *BAP1* alteration by conducting differential expression analysis between samples with a mutation (and/or CN loss) and samples with CN loss only, restricting analysis to a *BAP1* altered-only subset of five cancer types that have higher representation of mutations (>7 mutations): CHOL, LIHC, MESO, UVM, and KIRC. We computed a new per-sample *BAP1* mutation signature score (see Methods) based on the differentially expressed genes between samples with CN alterations alone and those with mutation alterations (Mut and CN+Mut) -- a combined total of 393 altered samples across the five cancer types (Figure 6A). Of 136 samples classified as mutation signature positive (score value ≥237), 80 were CN, 32 were CN+Mut, and 24 were Mut (Supplemental Table S7A). Mutation signature positive samples were enriched for samples that had a mutation (56/136, 41%) and were molecularly distinct compared to their mutation signature negative counterparts which were predominantly samples with copy number alterations (254/257, 99%).

We applied this *BAP1* mutational signature to all KIRC samples (including unaltered). The gene expression pattern differences between mutation signature positive (score value ≥139) and negative samples are less apparent but still separate those samples that carry mutations (13/13 mutant samples have positive mutation scores versus 64/323 CN samples with positive mutation scores, Figure 6B and Supplemental Table S3B). Further, *BAP1* mutation signature scores in KIRC sufficiently captured gene expression differences and separated unaltered, copy number only, and mutant samples (Figure 6C and Supplemental Table S3B). Gene set enrichment analysis revealed that the genes responsible for differences in mutation-driven versus copy number-driven *BAP1* alteration were broadly involved in metabolic processes (including oxidative phosphorylation), EMT, and immune-related functions (Figure 6D and Supplemental Table S7B). Therefore, wide-scale CN loss at chromosome 3p, is likely affecting many biological pathways amplified through co-occurring loss of multiple tumor suppressor genes including *PBRM1* and *VHL*, which are both frequent and well-known in KIRC.^49,50^ We stratified progression-free survival for KIRC based on positive and negative *BAP1* mutation signature scores and observed no difference in survival (Figure 6E, logrank p=0.5), in contrast to prior significant univariate and multivariate results using the more general *BAP1* alteration signature score (Table S6A-C).

## Discussion

### Improved classification of *BAP1* status in cancer

In this study, we used TCGA pan-cancer data from over 10,000 individuals to assess cancer type-specific prevalence as well as both shared and individual consequences of *BAP1* alteration. *BAP1* alterations – particularly those defined by mutations – have been studied in uveal melanoma, mesothelioma, and clear cell renal cell carcinoma but less so in other cancer types due in part to low occurrence of *BAP1* mutations.^1,15,11,51,2,13,12,16^ Here we report a 30% increase in detection of impactful *BAP1* somatic mutations across TCGA compared to prior MC3 mutation calls^24^ by using a *de novo* local realigner and merging variant calls from six individual callers across two pipelines (Figure S1 and Supplemental Table S2C). Among the new calls, 7 were indels ≥40bp, variant lengths that were previously undetected. This highlights that mutation calling pipelines continue to change and improve over time (there have been more than 20 data release updates from the GDC since the publication of the MC3 dataset in 2018) and that there is potentially great value in revisiting previous evaluations of gene mutations. Despite long read sequencing technology improving detection of larger indels and becoming more accessible, large historical datasets like TCGA that used short read sequencing will continue to benefit from new analyses utilizing software capable of detecting those indels.

In addition to mutations, we also expanded the alteration definition for *BAP1* by including gene-level CN loss. In mesothelioma, it was previously reported that, when adding copy number information and mutation pipelines that include methods to identify larger indels, the number of *BAP1*-altered samples increased by 40%.^16^ We show here that CN loss of *BAP1* is frequent in many cancer types: approximately 15% of pan-cancer samples carry some form of *BAP1* alteration, predominantly CN loss, compared to 1% carrying a mutation only (Supplemental Table S3A-B). 13/32 (41%) cancer types with *BAP1* alterations are entirely copy number-driven (Figure 2B). If accounting for mutations alone, alterations in these tumor types would be missed altogether and be excluded from downstream analyses. For other copy number-driven cancer types, the small number of mutations would similarly result in analyses being underpowered to examine differences in *BAP1*-altered versus -unaltered samples. One caveat is that copy number *BAP1* alterations may behave differently; for example, there were distinct gene expression patterns separating samples with *BAP1* mutations and those with copy number alterations (Figure 6A).

### Transcriptional profile changes associated with *BAP1* alteration

In UVM, there was a strong pattern of genes impacted by *BAP1* alteration which represented an array of biological changes that was mostly shared across cancer types (Figure 3D). Pathway-level analyses yielded results consistent with both known and proposed roles of *BAP1*, particularly in DNA repair^5,52^, immune response^53–55^, EMT^56,57^, apoptosis^9,10,58^, *Notch* signaling^59,60^, oxidative phosphorylation^61^, and cell cycle processes^1,6,7,15^ (Figure 3E). Variation in the direction and strength of pathway enrichment were likely due to a combination of cancer type-specific factors such as base-level expression differences across differentiated tissues as well as, importantly, co-altered gene interactions -- particularly as a result of arm-level CN loss. However, despite being CN-driven, GBM, OV, LGG, and HNSC are positively correlated with the UVM signature and are hierarchically clustered near each other at the hallmark pathway level (Figures 3D and 3E). Such CN-driven tumors that have expression patterns more similar to mutation-driven tumors may reflect a more *BAP1*-driven specific response.

In KIRC, which has both the highest overall frequency of CN loss and wide arm-level segments of loss (Figure 2A), there are other known renal tumorigenesis driver genes located on chromosome 3p which are functionally important frequently co-altered: *PBRM1*, *VHL*, and *SETD2*.^11,63–70^ This may partially explain why KIRC showed a negative correlation with the UVM-derived *BAP1* alteration signature (Figure 3D) and better progression-free survival outcomes when using the *BAP1* alteration signature but there was no survival difference when using a *BAP1* mutation-specific signature (Figures 5A and 6E).

### Role of *BAP1* in cell identity and differentiation

In hepatocellular carcinoma (LIHC), we recently characterized three molecular subsets of tumors based on integrated genomic characterization of transcriptional profiling, mutational signatures, and CN status.^14^ One of these subsets, cholangiocarcinoma-like, histologically classified as hepatocellular carcinoma but highly correlated to a defined cholangiocarcinoma mRNA expression centroid, was originally defined by IDH1 loss^71^ and we further showed it to have frequent *BAP1* loss as a major altered feature. Hepatocellular carcinoma tumors with altered *BAP1* had intermediate bile duct signature scores – higher than other LIHC tumors (Mann-Whitney U test Bonferroni adjusted p=3.7e-4) and lower than cholangiocarcinoma tumors (Figure 4D). Additionally, among the most positively enriched terms between *BAP1-*altered and -unaltered tumors in the MSigDB C8 database derived from single cell experiments were features defining bile duct cells whereas the most negatively enriched terms included hepatocytes and hepatoblasts (Figure 4C), suggesting that *BAP1*-altered tumors experience a transcriptional profile shift resulting in expression of prototypical cholangiocyte features.

These results highlight *BAP1* as an important coordinator in differentiation processes and maintenance of cell identity in cancer. This concept was explored experimentally in *Xenopus laevis* development wherein *BAP1* was shown to regulate lineage commitment through broad changes in transcriptional activation associated with H3K27ac.^72^ These changes resulted in failure to silence pluripotency factors and failure to induce expression of lineage-specific factors, leading to an aberrant and less-differentiated phenotype. The authors further relate these changes to their prior findings in uveal melanoma, where *BAP1* loss was associated with upregulation of pluripotency genes.^73^

Our findings emphasize the importance of implementing a multi-omic approach to detect *BAP1* alterations in order to better understand the consequences of *BAP1* loss promoting cancer development, cancer progression, and regulation of cell identity. We show that *BAP1* alteration affects similar pathways but in a cancer type-specific manner dependent on the tissue context and co-alteration landscape.

## Supplementary Material

Supplement 1

Supplement 2

Supplement 3

Supplement 4

Supplement 5

Supplement 6

Supplement 7

Supplement 8
