# Supplementary material for: Expanded detection of BAP1 alterations in cancer and tumor type-specific expression score comparison
Source: bioRxiv. 2023 Nov 21:2023.11.21.568094. Preprint. [Version 1] doi: 10.1101/2023.11.21.568094 (PMC10690206; doi:10.1101/2023.11.21.568094)

bioRxiv preprint doi: https://doi.org/10.1101/2023.11.21.568094; this version posted November 21, 2023. The copyright holder for this preprint (which was not certified by peer review) is the author/funder, who has granted bioRxiv a license to display the preprint in perpetuity. It is made available under aCC-BY 4.0 International license.

Supplemental

Supplemental Figure S1. *BAP1* variant lengths for variants detected across mutation calling pipelines, comparing the prior TCGA MC3 dataset (n=92 variants) and the new dataset (n=130 variants) which combined updated TCGA GDC variant calls with calls from an ABRA2/Cadabra/Strelka2 workflow. Blue dotted line indicates the threshold for variant lengths ≥40bp.

Supplemental Figure S2. **(A)** Variant allele frequency for *BAP1* variants in pan-cancer mutant samples, separated by alteration type. P-value derived from two-sided Mann-Whitney U test with continuity correction. **(B)** Scatterplot of tumor purity and variant allele frequency for *BAP1* variants in pan-cancer mutant samples, colored by sample alteration type. Dotted line represents identity (y=x) line. Adjusted r-squared value derived from linear regression in R which performs QR decomposition followed by Wherry adjustment. Mut: mutation only, CN+Mut: gene-level copy number loss and mutation.

Supplemental Figure S3. Pan-cancer *BAP1* RNA-level expression by alteration type. P-values derived from pairwise two-sided Mann-Whitney U test with continuity correction and Bonferroni adjustment. CN: gene-level copy number loss, Mut: mutation only, CN+Mut: gene-level copy number loss and mutation.

Supplemental Figure S1

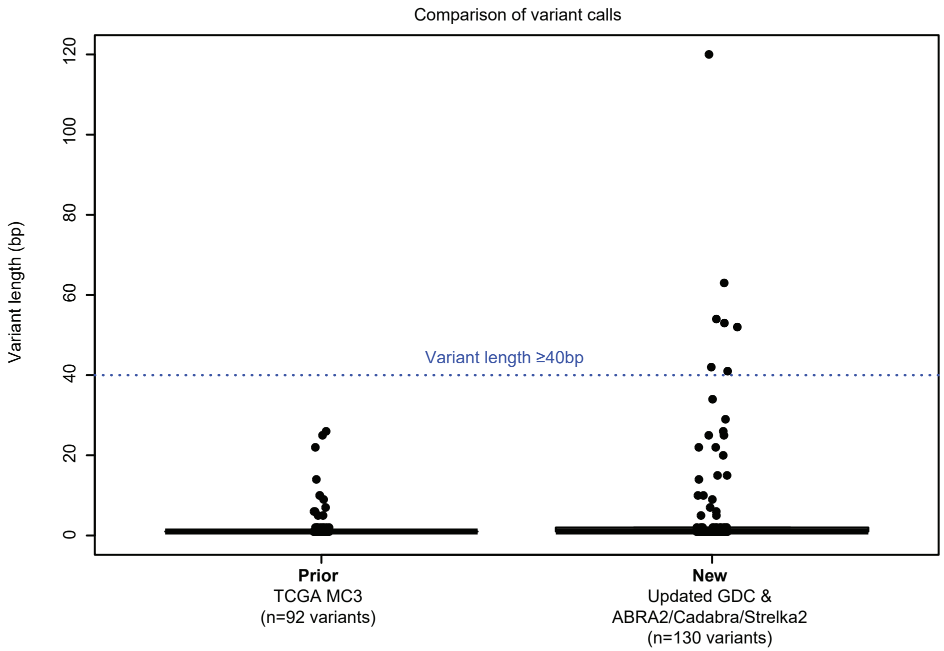

Supplemental Figure S2

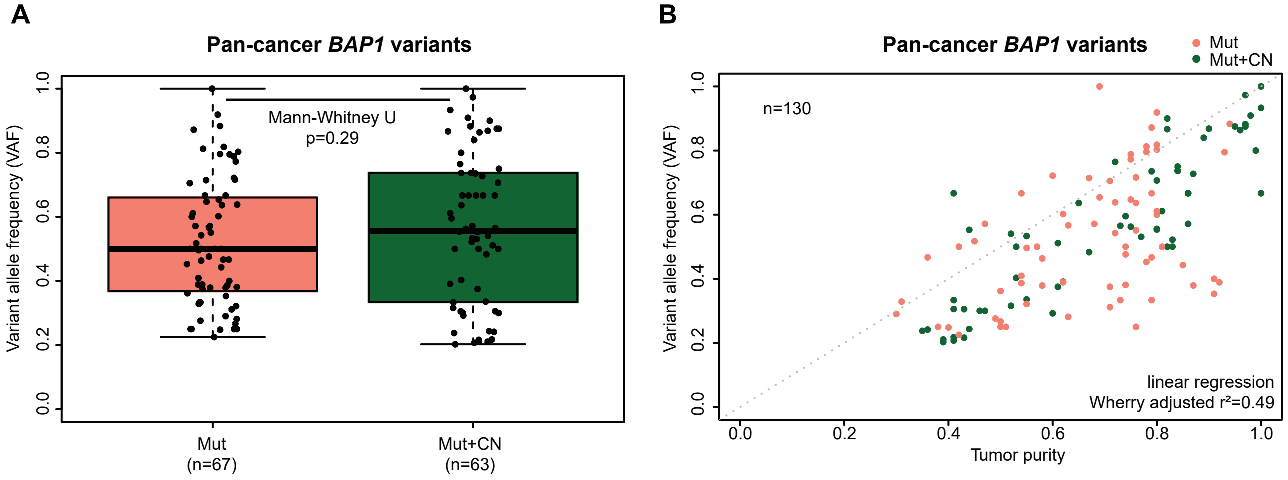

Supplemental Figure S3

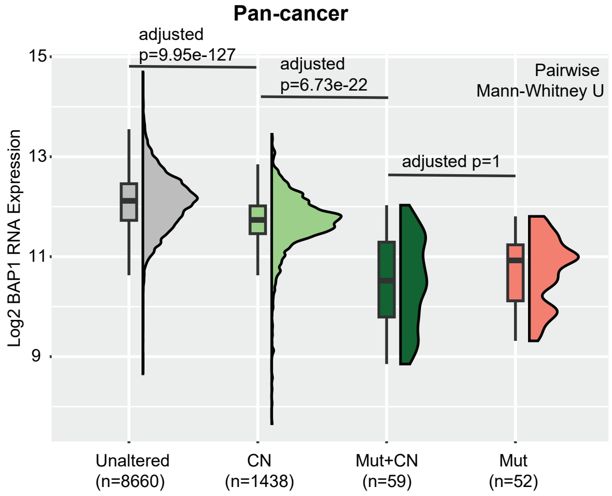

Supplement: Supplement 8 [file NIHPP2023.11.21.568094V1-supplement-8.pdf]
